# Application of mind map can promote the health education effect of children with vasovagal syncope

**DOI:** 10.3389/fcvm.2023.1051677

**Published:** 2023-02-16

**Authors:** Ping Liu, Wanzhen Mei, Mengying Zhou, Ting Zhao, Yuwen Wang, Runmei Zou, Cheng Wang

**Affiliations:** ^1^Clinical Nursing Teaching and Research Section, The Second Xiangya Hospital, Central South University, Changsha, Hunan, China; ^2^Department of Pediatric Cardiovasology, Children's Medical Center, The Second Xiangya Hospital, Central South University, Changsha, Hunan, China

**Keywords:** children, vasovagal syncope, health education, effect, mind map

## Abstract

**Objective:**

To explore the effect of mind map on health education in children with vasovagal syncope (VVS).

**Methods:**

In this prospective controlled study, 66 children with VVS (29 males, 10.38 ± 1.80 years) and their parents (12 males, 39.27 ± 3.74 years) who were hospitalized in the Department of Pediatrics, The Second Xiangya Hospital, Central South University from April 2020 to March 2021 were set as the control group. 66 children with VVS (26 males, 10.29 ± 1.90 years) and their parents (9 males, 38.65 ± 1.99 years) who were hospitalized in the same hospital from April 2021 to March 2022 were set as the research group. Traditional oral propaganda method was applied in the control group, and the health education method based on mind map was applied in the research group. The self-designed VVS health education satisfaction questionnaire and comprehensive health knowledge questionnaire were used to conduct on-site return visits to the children and their parents who had been discharged from the hospital for 1 month.

**Results:**

There was no significant difference in age, sex, hemodynamic type of VVS, and the parental age, sex, education level between the control group and the research group (*P* > 0.05). Health education satisfaction score, health education knowledge mastery score, compliance score, subjective efficacy and objective efficacy in the research group were higher than those in the control group (*P* < 0.05). If the satisfaction score, knowledge mastery score, and compliance score increase by 1 point, the risk of poor subjective efficacy is reduced by 48, 91, and 99%, respectively, and the risk of poor objective efficacy is reduced by 44, 92, and 93%, respectively.

**Conclusions:**

Application of mind map can improve the health education effect of children with VVS.

## 1. Introduction

Vasovagal syncope (VVS) is a common type of neurally mediated syncope in children, and the incidence is higher in females than males (1–4). It is characterized by transient loss of consciousness, inability to maintain an upright posture of the body and falling to the ground. Clinically, some patients may have recurrent syncope episodes or pre-syncope symptoms, and even syncope-related physical accidental injury (5, 6), or anxiety and depression, loss of appetite, increased somatic symptoms and loss of confidence, resulting in a decrease in the quality of life of the child and seriously affecting the patient's learning and life (7). Taking effective intervention measures for VVS as early as possible can avoid or reduce the onset of these symptoms. Clinically, the treatment of VVS mainly based on non-drug treatment such as health education (8), including avoiding predisposing factors, anti-resistance training and increasing water and salt intake (8–10), etc. Syncope and pre-syncope symptoms can be effectively reduced through health education and some lifestyle changes (e.g., recognizing syncope prodromal symptoms, increasing water intake, anti-resistance training and physical exercise, etc.), thereby improving the quality of life and learning for children with VVS (11). It can be seen that effective health education and lifestyle guidance are crucial in VVS rehabilitation. At present, the health education methods for children with VVS are mostly dependent on traditional oral education. Children with VVS who were hospitalized for treatment were short in hospital, and the communication and expression skills of the mission nurses were different, the effective health education information received by children and their parents was limited, resulting in the failure of health education. In order to explore effective ways to improve the quality and efficiency of health education for children with VVS, this study introduced the health education method of mind map.

The mind map is a thinking tool that concretizes divergent thinking. It uses lines, symbols, vocabulary and images to form divergent and node-like structural forms, and turns cumbersome text information into a visual diagram with clear layers and rich pictures and texts. It not only omits the tedious and complicated oral communication process, but also intuitively provides visual stimulation to children and their parents, deepens the impression of key content, and eliminates the inefficient information dissemination caused by hearing fatigue, and allows learners to store and extract information more effectively, and improve work and learning efficiency (12, 13). Some studies have shown that mind maps can improve the health education effect of patients (14–16). Li et al. (17) has found that the combined communication mode of SBAR (namely Situation, Background, Assessment, Recommendation) standard and mind map used in the emergency department can improve the quality of handover, reduce adverse events and handover problems, and get higher patient satisfaction. In this study, mind map was introduced to the health education of children with VVS and their parents, and the effect of mind mapping on VVS children's health education was observed.

## 2. Study population and methods

### 2.1. Study population and data collection

**Figure 1:**
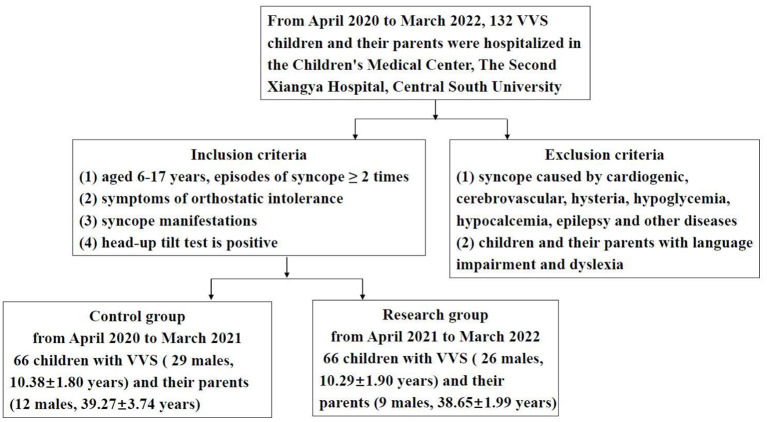
Inclusion criteria and exclusion criteria.

A prospective controlled study was conducted. The inclusion criteria and exclusion criteria were shown in Figure 1. Children with VVS and their parents were divided into control group and research group. The study subjects returned to the hospital for follow-up visits 1 month after discharge.

The sample size was calculated by using the formula *n*1 = *n*2 = [(*Z*α + *Z*β)^2^ × 2σ^2^]/δ^2^. The outcome index of this study was compliance score. The average compliance score of the control group was expected to be 3.00 ± 1.3 points, and the compliance score of the study group was expected to be increased by 1.0 points. Bilateral test, α was 0.05, the sample size ratio of the two groups was 1:1, and the test efficacy was 1 – β = 90%. The sample size calculated was 41. Consider a 25% shedding rate, the required minimum total number of participants was 51 in our study. We selected a sample size of 66 cases in each group.

### 2.2. Implementer of health education

The health education of the research group and the control group were implemented by pediatric specialist nurses, aged 26–45 years old, with an average age of 33.00 ± 5.03 years old.

### 2.3. Methods of health education

**Figure 2:**
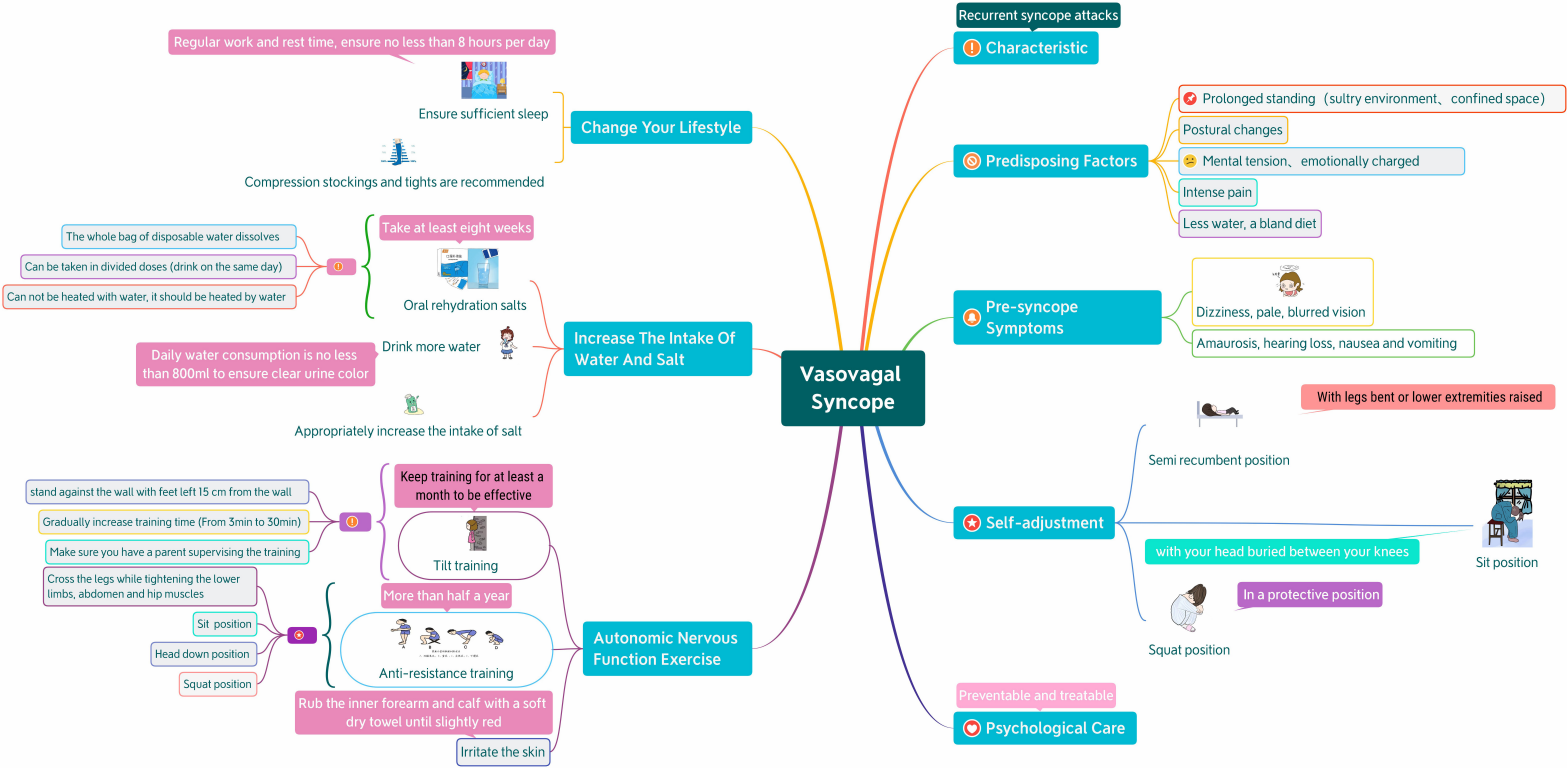
Mind map of health education for children with vasovagal syncope and their parents.

The control group received the conventional health education method, namely the oral education method, which lasted about 15–30 min. After the diagnosis was confirmed, the nurses explain VVS-related knowledge to the children and their parents, including common predisposing factors for syncope, increasing intake of water and salt, methods of taking oral rehydration salts, and instructing children on autonomic nerve function exercises, emphasizing that exercises should be performed as required. The research group received the mind map health education method, about 10–15 min. First of all, the nurses in our hospital would be trained on the knowledge of mind map. The training content was based on the medical knowledge of VVS, focusing on the background and usage of mind map. A total of 4 h of training would be conducted. After passing the real-life operation assessment, they would participate in this study. On the basis of evidence-based nursing and clinical experience, the members of the research team made a mind map, based on the content of traditional VVS health education and guided by the general information of children and their parents, to select the key points of pediatric VVS health education, from the perspective of patients, consult the literature and design a mind map of pediatric VVS self-prevention management based on peer practices. The drawing follows the principles of reasonable logic, clear fonts, and rich pictures and texts, and uses the mind map software X Mind 8 to draw (Figure 2). After the diagnosis was confirmed, the children and their parents were given health education by using mind maps. The nurses emphasized the key points one by one according to the mind maps, and guided the children to exercise their autonomic nerve function. After the teaching, hand over the mind map to the children and their parents, and guide them to consolidate knowledge and improve compliance as shown in the picture. Nurses supervise their implementation in their daily work, and carry out targeted health education as appropriate.

### 2.4. Evaluation method

#### 2.4.1. General information questionnaire

The research team asked children and their parents to complete the questionnaire. The contents of the questionnaire included the child's name, sex, date of birth, academic performance, place of residence and parents' age, education level, and telephone number.

#### 2.4.2. Health education satisfaction questionnaire (for children and their parents)

A self-designed health education satisfaction questionnaire was used. The contents include nurses' introduction of disease-related knowledge, guidance on eating and living habits, guidance on autonomous function exercise, guidance on drugs and psychological counseling. The score calculation standard was as follows: 2, satisfaction; 1, general; 0, dissatisfaction. The full score is 16 scores. The higher the score, the higher the satisfaction. The assessment was performed by the patient and the parents before the patient was discharged from the hospital. We collected 66 valid questionnaires from the research group and the control group respectively, and the recovery rate and effective rate were both 100%.

#### 2.4.3. Health education knowledge questionnaire

The self-designed VVS health education knowledge questionnaire was used to conduct on-site return visits to the children and their parents who had been discharged from the hospital for 1 month, and understand their mastery of VVS health education knowledge, syncope frequency, compliance and re-examination of head-up tilt test (HUTT) results, etc. The specific content is as follows: (1) What are the predisposing factors for syncope? (At least answer 3 or more predisposing factors, such as prolonged standing, postural changes, strenuous exercise, etc.). (2) What to do if fainting occurs? (For example, keep your supine position with your legs slightly elevated; in a sitting position, bury your head between your knees; in a squat position, clasp your hands tightly and hold your legs tightly in a protective position). (3) What are the precautions for oral rehydration salts? (The daily amount is dissolved at one time and can be taken in divided doses; it cannot be mixed with water in the middle; it can be warmed with water; drink it up on the same day). (4) How is the autonomic nervous system exercise performed? (Such as stand training, anti-resistance training, etc. Answer at least one of them and be absolutely correct). (5) Are you taking oral rehydration salts as prescribed by your doctor? (6) Do you do autonomic nervous system exercise every day? (7) Are you guaranteed three meals a day and enough sleep? (Sleep at least 8 h per day). (8) Do you use compression stockings or tights pants? In the questionnaires survey, “know” is scored as 1, “don't know” is scored as 0; if the answer is “yes,” it is scored as 1, and the answer of “no” is scored as 0. 66 valid questionnaires were collected from the research group and the control group, respectively, and the recovery rate and effective rate were both 100%.

#### 2.4.4. Evaluation of efficacy

(1) Subjective efficacy: after training, the number of syncope or the occurrence of syncope premonitory in the child was reduced compared with that before treatment or had no episodes, which was regarded as improvement; after the training, the number of syncope or the occurrence of syncope is increased or no change, which was regarded as noimprovement. Efficiency (Improvement rate) = number of improved cases/total number of cases. (2) Objective efficacy: according to the HUTT results of the patient's re-examination, the HUTT induced syncope time was longer than the previous once, or the result change from positive to negative, which was regarded as improved; shortened time to induction of syncope, or no change in HUTT results was considered no improvement.

#### 2.4.5. Head-up tilt test (HUTT)

##### 2.4.5.1. Preparations before the test

Children fasted for at least 4 h before the testing, any vasoactive medication was stopped for at least five half-lives, and drink that could affect autonomic nervous system function (e.g., coffee) was avoided. The test was performed ata suitable temperature, dim light, and quiet environment. Detailed instructions and risks should be described to children and their legal guardians/parents. Signed informed consent should be obtained before the test (9, 18, 19).

##### 2.4.5.2. Basic HUTT (BHUT)

The test was performed using Head-up Tilt Test Mornitoring System (SHUT-100) of Jiangsu Standard Medical Technology Co., Ltd. after overnight fasting between 8:00 a.m. and 12:00 p.m. The subjects were asked to lay still for 10 min, and then baseline heart rate (HR), blood pressure (BP), and ECG were recorded. Subjects were tilted at 60° with head upward, and HR, BP, and ECG were recorded continuously until either 45 min duration, or the development of syncope or intolerable near syncope symptoms. If syncope occurred, patients were rapidly placed in the supine position.

##### 2.4.5.3. Sublingual nitroglycerin HUTT (SNHUT)

If the subjects did not develop syncope or pre-syncope, they underwent SNHUT. A tilted posture was maintained, subjects were medicated with nitroglycerin 4–6 μg/kg (maximum ≤ 300 μg), and HR, BP, and ECG were recorded for 20 min or until syncope or pre-syncope occurred.

##### 2.4.5.4. Diagnosis of VVS and response types

VVS was defined as the development of syncope or pre-syncope accompanied by hypotension (systolic BP ≤ 80 mmHg and/or diastolic BP ≤ 50 mmHg, or over 25% decrease in mean BP), bradycardia (HR < 75 bpm for children at 4–6 years of age, <65 bpm for children at 6–8 years of age, and <60 bpm for children at 8 years of age and older), or cardiac arrest >3 s. VVS was further classified into three responses: vasoinhibitory type VVS (VVS-VI) (significant reduction in BP but insignificant change in HR), cardioinhibitory type VVS (VVS-CI) (significant reduction in HR but insignificant change in BP), and mixed type VVS (VVS-M) (significant reduction both in BP and HR).

### 2.5. Statistical analysis

The continuous variables were characterized by mean ± standard deviation, or as median and interquartile range, as appropriate. The categorical variables were presented as a number (*n*) and percentages (%). The Student's *t*-test, Chi-square test, Fisher's exact test, or Mann–Whitney *U* test were conducted to compare factors between two groups, as appropriate. Univariate binary Logistic regression was used to briefly evaluate the approximate effects of factors on the efficacy and direction of both subjective efficacy and objective efficacy. Multifactor Logistic regression to analyze the possible association between subjective efficacy and objective efficacy and many factors was used and constructed two models to illustrate the stability of this relationship: Model 1 adjusted for sociodemographic data (sex, age); Mode 2 adjusted for sociodemographic data and parental sex and age and education level, hemodynamic type of VVS. All the analyses were performed with the statistical software packages R (version 3.6.1) (http://www.R-project.org, The R Foundation) and EmpowerStats (http://www.empowerstats.com, X & Y Solutions, Inc, Boston, MA). *P*-values < 0.05 (two-sided) were considered statistically significant.

## 3. Results

### 3.1. General data and questionnaire data analysis

There were no significant differences in age and sex of the children and their parents between the research group and the control group (*P* > 0.05). The satisfaction on health education, the awareness rate of health education, the compliance of health education, the satisfaction score, the knowledge score, the compliance score, the subjective efficacy and the objective efficacy of the research group were higher than those of the control group (*P* < 0.01). There was no statistical difference in hemodynamic types of VVS and parental education between the research group and the control group (*P* > 0.05) (Table 1).

**Table 1:** General data of the study population and description of questionnaire data [Mean ± SD/*n* (%)].

**Group**	**Control group (*n* = 66)**	**Research group (*n* = 66)**	**Standardize diff**.	***P*-value**
**Comparison of general data between the research group and the control group**				
Age (years)	10.38 ± 1.80	10.29 ± 1.90	0.05 (−0.29, 0.39)	0.778
Sex			0.09 (−0.25, 0.43)	0.596
Male	29 (43.94%)	26 (39.39%)		
Female	37 (56.06%)	40 (60.61%)		
Parents' age (years)	39.27 ± 3.72	38.65 ± 1.99	0.21 (−0.13, 0.55)	0.234
Parents' sex			0.12 (−0.22, 0.47)	0.475
Male	12 (18.18%)	9 (13.64%)		
Female	54 (81.82%)	57 (86.36%)		
Parents' education level			0.22 (−0.12, 0.56)	0.453
Middle school and below	36 (54.55%)	29 (43.94%)		
Senior middle school	20 (30.30%)	26 (39.39%)		
Undergraduate and above	10 (15.15%)	11 (16.67%)		
**Comparison of satisfaction with health education between the research group and the control group**				
The nurse explained to me how syncope happens			0.54 (0.19, 0.89)	0.008
Dissatisfaction	8 (12.12%)	0 (0.00%)		
General	17 (25.76%)	16 (24.24%)		
Satisfaction	41 (62.12%)	50 (75.76%)		
The nurse introduced me to pre-syncope symptoms			0.74 (0.39, 1.09)	<0.001
Dissatisfaction	6 (9.09%)	0 (0.00%)		
General	25 (37.88%)	11 (16.67%)		
Satisfaction	35 (53.03%)	55 (83.33%)		
The nurse explained to me what to do if I have fainting			0.90 (0.54, 1.26)	<0.001
Dissatisfaction	8 (12.12%)	0 (0.00%)		
General	20 (30.30%)	5 (7.58%)		
Satisfaction	38 (57.58%)	61 (92.42%)		
The nurse taught me how to take oral rehydration salts			0.45 (0.11, 0.80)	0.037
Dissatisfaction	4 (6.06%)	0 (0.00%)		
General	15 (22.73%)	9 (13.64%)		
Satisfaction	47 (71.21%)	57 (86.36%)		
The nurse instructed me on how to exercise the autonomic nervous function			0.53 (0.18, 0.88)	0.009
Dissatisfaction	8 (12.12%)	0 (0.00%)		
General	12 (18.18%)	12 (18.18%)		
Satisfaction	46 (69.70%)	54 (81.82%)		
The nurse guided me on my life and hygiene habits			0.50 (0.15, 0.84)	0.016
Dissatisfaction	7 (10.61%)	0 (0.00%)		
General	11 (16.67%)	10 (15.15%)		
Satisfaction	48 (72.73%)	56 (84.85%)		
The nurse guided my diet			0.57 (0.23, 0.92)	0.004
Dissatisfaction	8 (12.12%)	0 (0.00%)		
General	6 (9.09%)	12 (18.18%)		
Satisfaction	52 (78.79%)	54 (81.82%)		
The nurse eased my anxiety about the disease			0.56 (0.21, 0.91)	0.007
Dissatisfaction	5 (7.58%)	0 (0.00%)		
General	21 (31.82%)	12 (18.18%)		
Satisfaction	40 (60.61%)	54 (81.82%)		
**Comparison of the health education knowledge mastery between the research group and the control group**				
What are the predisposing factors for syncope?			0.55 (0.20, 0.89)	0.002
Don't know	11 (16.67%)	1 (1.52%)		
Know	55 (83.33%)	65 (98.48%)		
What to do if fainting occurs?			0.77 (0.41, 1.12)	<0.001
Don't know	15 (22.73%)	0 (0.00%)		
Know	51 (77.27%)	66 (100.00%)		
What are the precautions for oral rehydration salts?			0.63 (0.28, 0.98)	<0.001
Don't know	11 (16.67%)	0 (0.00%)		
Know	55 (83.33%)	66 (100.00%)		
How is the autonomic nervous system exercise performed?			0.92 (0.57, 1.28)	<0.001
Don't know	22 (33.33%)	1 (1.52%)		
Know	44 (66.67%)	65 (98.48%)		
**Comparison of compliance with health education between the research group and the control group**				
Are you taking oral rehydration salts as prescribed by your doctor?			0.87 (0.51, 1.22)	<0.001
No	18 (27.27%)	0 (0.00%)		
Yes	48 (72.73%)	66 (100.00%)		
Do you do autonomic nervous system exercise every day?			0.74 (0.39, 1.09)	<0.001
No	24 (36.36%)	5 (7.58%)		
Yes	42 (63.64%)	61 (92.42%)		
Are you guaranteed three meals a day and enough sleep?			0.67 (0.32, 1.02)	<0.001
No	12 (18.18%)	0 (0.00%)		
Yes	54 (81.82%)	66 (100.00%)		
Do you use compression stockings or tights pants?			0.52 (0.18, 0.87)	0.004
No	16 (24.24%)	4 (6.06%)		
Yes	50 (75.76%)	62 (93.94%)		
**Comparison of satisfaction scores, knowledge mastery scores, and compliance scores between the research group and the control**				
**group**				
Satisfaction scores	12.44 ± 3.08	14.68 ± 1.60	0.91 (0.56, 1.27)	<0.001
Knowledge mastery scores	3.09 ± 1.09	3.97 ± 0.17	1.12 (0.76, 1.49)	<0.001
Compliance scores	2.91 ± 1.17	3.86 ± 0.46	1.07 (0.71, 1.44)	<0.001
**Comparison of subjective and objective efficacy between the research group and the control group**				
Subjective efficacy			0.59 (0.25, 0.94)	0.001
Improved	48 (72.73%)	62 (93.94%)		
No improved	18 (27.27%)	4 (6.06%)		
Objective efficacy			0.61 (0.26, 0.96)	<0.001
Improved	36 (54.55%)	54 (81.82%)		
No improved	30 (45.45%)	12 (18.18%)		
**Comparison of hemodynamic type of VVS between the research group and the control group**				
VVS hemodynamic types			0.17 (-0.17, 0.52)	0.639
Vasoinhibitory type	52 (78.79%)	49 (74.24%)		
Cardioinhibitory type	2 (3.03%)	1 (1.52%)		
Mixed type	12 (18.18%)	16 (24.24%)		

### 3.2. Univariate analysis of subjective efficacy and objective efficacy

The research group was the protective factor of subjective efficacy and objective efficacy. The ages of the children and their parents were the risk factors of subjective efficacy, the education level of the parents in high school was the protective factor of subjective efficacy and objective efficacy, the satisfaction score and the knowledge mastery score and the compliance score were the protective factor of subjective efficacy and objective efficacy (*P* > 0.05) (Table 2).

**Table 2:** Univariate analysis of subjective efficacy and objective efficacy [*n* = 132, Mean ± SD/*n* (%)].

	**Statistics**	**Subjective efficacy**		**Objective efficacy**	
		**OR (95%CI)**	***P*** **-value**	**OR (95%CI)**	***P*** **-value**
**Group**					
Control group	66 (50.00%)	1.0		1.0	
Research group	66 (50.00%)	0.17 (0.05, 0.54)	0.003	0.27 (0.12, 0.59)	0.001
Age (years)	10.33 ± 1.84	1.39 (1.08, 1.79)	0.012	1.26 (1.02, 1.55)	0.029
**Sex**					
Male	55 (41.67%)	1.0		1.0	
Female	77 (58.33%)	1.04 (0.41, 2.63)	0.937	1.67 (0.78, 3.59)	0.187
Parents' age (years)	38.96 ± 2.99	1.20 (1.03, 1.40)	0.022	1.12 (0.98, 1.27)	0.088
**Parents' sex**					
Male	21 (15.91%)	1.0		1.0	
Female	111 (84.09%)	0.42 (0.14, 1.25)	0.118	0.56 (0.22, 1.47)	0.240
**Parents' education level**					
Middle school and below	65 (49.24%)	1.0		1.0	
Senior middle school	46 (34.85%)	0.88 (0.31, 2.48)	0.810	0.47 (0.20, 1.12)	0.090
Undergraduate and above	21 (15.91%)	1.16 (0.33, 4.10)	0.824	1.05 (0.38, 2.90)	0.923
**Hemodynamic type of VVS**					
Vasoinhibitory type	101 (76.52%)	1.0		1.0	
Cardioinhibitory type	3 (2.27%)	2.87 (0.24, 33.63)	0.402	1.18 (0.10, 13.55)	0.892
Mixed type	28 (21.21%)	1.56 (0.54, 4.50)	0.407	1.53 (0.64, 3.66)	0.337
Satisfaction scores	13.56 ± 2.69	0.71 (0.60, 0.85)	0.000	0.67 (0.56, 0.79)	0.000
Knowledge mastery scores	3.53 ± 0.89	0.21 (0.12, 0.40)	0.000	0.12 (0.05, 0.27)	0.000
Compliance scores	3.39 ± 1.01	0.04 (0.01, 0.15)	0.000	0.10 (0.05, 0.23)	0.000

Data in table: OR, odds ratio; CI, confidence interval.

Result variable: subjective efficacy, objective efficacy.

Exposure variable: group, sex, parents' sex, hemodynamic type of VVS, parents' education level, age, parents' age, satisfaction scores, knowledge mastery scores, compliance scores.

Adjusted variable: none.

### 3.3. Comparison of multifactor regression equations of subjective efficacy and objective efficacy

In order to further clarify whether satisfaction score, knowledge mastery score and compliance score had independent and stable influences on subjective and objective efficacy in univariate analysis, we conducted a comparison of multifactor binary Logistic regression equations. After adjusting different confounding factors, the model still showed good stability and small fluctuation of effect value. The satisfaction score, knowledge mastery score, and compliance score increased by 1 point, the risk of poor subjective efficacy was reduced by 48, 91, and 99%, respectively, and the risk of poor objective efficacy was reduced by 44, 92, and 93%, respectively (Table 3).

**Table 3:** Comparison of multifactor regression equations of subjective efficacy and objective efficacy.

**Exposure**	**Model 1**		**Model 2**	
	**OR (95% CI)**	***P*** **-value**	**OR (95% CI)**	***P*** **-value**
**Subjective efficacy**				
Satisfaction scores	0.66 (0.54, 0.82)	0.000	0.52 (0.36, 0.74)	0.000
Knowledge mastery scores	0.18 (0.09, 0.36)	0.000	0.09 (0.03, 0.27)	0.000
Compliance scores	0.02 (0.00, 0.15)	0.000	0.01 (0.00, 0.13)	0.000
**Objective efficacy**				
Satisfaction scores	0.65 (0.54, 0.79)	0.000	0.56 (0.43, 0.73)	0.000
Knowledge mastery scores	0.12 (0.05, 0.29)	0.000	0.08 (0.03, 0.23)	0.000
Compliance scores	0.09 (0.03, 0.21)	0.000	0.07 (0.03, 0.19)	0.000

Data in table: OR, odds ratio; CI, confidence interval.

Result variable: subjective efficacy, objective efficacy.

Exposure variable: satisfaction scores, knowledge mastery scores, compliance scores.

Model 1 adjusted for: sex, age.

Model 2 adjusted for: sex, age, parents' sex, parents' age, hemodynamic type of VVS, parents' education level.

## 4. Discussion

VVS is a common neurally mediated syncope in clinical practice, and health education is the basic measure for intervention of VVS (8). Although the prognosis of VVS is good, due to the lack of knowledge of the disease and health education of the children and their parents, the compliance of the intervention and the rehabilitation effect are affected, which can lead to the recurrence of syncope or pre-syncope. In addition to seriously affecting the physical and mental health and quality of life of the children, the parents are also under great psychological pressure. Therefore, paying attention to the psychological feelings and service requirements of children with VVS and their parents, especially the health education of children and their parents, and improving their compliance, are the key links to promoting the rehabilitation of children (20, 21).

The purpose of health education is not only to “educate knowledge,” but also to establish “medical compliance behavior or healthy behavior” through guidance. Its effect is closely related to factors such as health educators, health education methods and health education objects. In order to avoid the omission of information or unclear expression due to the differences in theoretical knowledge and language expression ability of health educators, the health education practitioners in this study are all highly educated nurses who have undergone unified training and have worked in clinical practice for at least 3 years. Individuals have different abilities to understand and accept information, and people have limited short-term memory, and the content of oral education is easy to forget, which may lead to children and their parents lack of information acceptance or lack of understanding, resulting in reduced compliance. The mind map used in this study can make up for this deficiency. Mind map can turn boring information into colorful and well-organized diagrams to help understand memory (13, 22) (Figure 2).

### 4.1. Health education method based on mind map can improve the satisfaction of children with VVS and their parents with health education

The results of this study showed that the research group's satisfaction score for health education was increased compared with the control group (*P* < 0.01), and the risk of poor subjective efficacy was decreased by 48% and the risk of poor objective efficacy was decreased by 44% for each point increase of satisfaction score of health education in clinical nursing work in the research group. It is suggested that the health education method based on mind mapping can improve the satisfaction of health education and intervention effect of children with VVS and their parents. Zhang et al. (23) applied mind map to the health education of parents of children with infectious mononucleosis, and found that the research group was significantly higher than the control group in terms of nursing satisfaction in nurse-patient communication, health education, and nursing technology (*P* < 0.01). This may be related to the simplicity and clarity of the mind map, focusing on prominent features, which is more conducive to the transmission of information. Targeted health education can promote emotional communication between nurses and patients, increase the trust between nurses and patients, and thus improve the satisfaction of children and their parents with clinical nursing work. Gakhal et al. (13) also reported that the use of audiovisual presentations and mind map exercises to improve the information recall ability of orthodontic patients and their parents and found that the use of audiovisual presentations and written information can improve the information recall rate of orthodontic patients and their parents, and thought mind map is a better form than written information. It can be seen that the health education method based on mind mapping can improve the health education effect of children with VVS and their parents.

### 4.2. The health education method based on mind map can improve the mastery of health education knowledge and compliance in children with VVS and their parents

Through the health education knowledge questionnaire, it was found that the research group had significantly higher scores on the mastery of health education knowledge and compliance than the control group (*P* < 0.01). And in research group, the risk of poor subjective efficacy was decreased by 91 and 99% for each point increase of the score of mastery knowledge and compliance, respectively. The risk of poor objective efficacy was decreased by 92 and 93% for each point increase of the score of mastery knowledge and compliance, respectively. The above results suggested that the health education based on mind map can improve the compliance of children with VVS and their parents to health education, avoid possible clinical inducements and triggers, effectively increase the intake of water and salt, and ensure the effectiveness of the intervention. Studies have shown that the application of mind map in pediatric VVS health education can improve the compliance of children and their parents. Sesanelvira et al. (24) reported the educational effect of using mind map method on school-age children's food safety behavior, and found that the use of mind map method has a significant impact on school-age children's food safety knowledge, attitudes and skills. It is believed that the mind map method can be applied to improve the clean and healthy living behaviors of school-age children. Yang et al. (25) reported the application of mind map health education in extended care for children with caries, and found that the use of mind map health education method could significantly improve the caries knowledge of children with caries and their parents, and improve their compliance to health education. Therefore, mind mapping is an appropriate health education tool that can be used for extended care of children with dental caries. In conclusion, health education based on mind map can carry out effective cognitive and behavioral intervention for children and their parents, thereby significantly improving the cognitive level of children and their parents in dealing with disease-related knowledge, and effectively improving the compliance and quality of life of children.

### 4.3. The health education method based on mind map can improve the subjective efficacy and objective efficacy of VVS children

In this study, both the subjective efficacy and objective efficacy of the research group and the control group were improved, and the subjective and objective efficacy of the research group were better than those of the control group (*P* < 0.01). And the research group was the protective factor of subjective efficacy and objective efficacy, the satisfaction score, the knowledge mastery score and the compliance score were the protective factor of subjective efficacy and objective efficacy, which was related to the improvement of patient compliance behavior with mind map (26). The mind map is easy to understand and can make up for the unclear explanations and understandings in oral health education. It can carefully let children with VVS and their parents clarify the rehabilitation knowledge of VVS, and provide guidance for effective rehabilitation and improved prognosis. This study shows that the parents' education level in high school is a protective factor for both subjective efficacy and objective efficacy, indicating that the group with higher education levels has a higher acceptance level and understanding ability of health education, which is conducive to improving compliance. The ages of the children and their parents were risk factors for subjective efficacy, which may be related to the obvious anxiety about syncope or pre-syncope episodes in older age.

It can be seen that the application of a mind map in the health education of children with VVS and their parents can increase the clinical intervention effect.

## 5. Shortcomings and prospects

However, this study had certain limitations. It was a single-center-based research, the subjects were younger and the sample size was not large enough. At the same time, due to the impact of the COVID-19 epidemic, the research group did not recruit the research subjects at the same time, which needs to be avoided in the future research. Therefore, further large sample and multi-center studies can be conducted in the future, and the outcome indicators can be added to verify the educational program. It is recommended to promote and establish patient health records in pediatric cardiovascular outpatient clinics, record the progress of health education during patient follow-up, and lay a foundation for follow-up research.

## Data availability statement

The original contributions presented in the study are included in the article/supplementary material, further inquiries can be directed to the corresponding author.

## Ethics statement

The studies involving human participants were reviewed and approved by the Ethics Committee of The Second Xiangya Hospital, Central South University. Written informed consent to participate in this study was provided by the participants' legal guardian/next of kin.

## Author contributions

PL and WM had primary responsibility for the protocol development, patient enrollment, data collecting preliminary data analysis, and writing the manuscript. MZ was responsible for the drawing. TZ was responsible for language polishing. YW and RZ completed the head-up tilt test. PL and CW assisted with critical revision for important content and edited the draft. CW supervised the design, execution of the study, checked the data analysis, and contributed to a final approval of the manuscript submitted. All authors have read and approved the final manuscript and assumed full responsibility for its contents.
